# Breast cancer survival and incidence of second primary cancers after 30 years in a randomized study of two versus five years of adjuvant tamoxifen therapy

**DOI:** 10.1016/j.breast.2023.07.010

**Published:** 2023-07-25

**Authors:** Anna Nordenskjöld, Helena Fohlin, Johan Rosell, Nils-Olof Bengtsson, Tommy Fornander, Thomas Hatschek, Henrik Lindman, Per Malmström, Lisa Rydén, Arne Wallgren, Olle Stål, Bo Nordenskjöld

**Affiliations:** a Department of Medicine and Oncology, Southern Älvsborg Hospital, 50182, Borås, Sweden; b Department of Oncology, Institute of Clinical Sciences, Sahlgrenska Academy, Sahlgrenska University Hospital, 413 45, Gothenburg, Sweden; c Regional Cancer Center Southeast Sweden and Department of Biomedical and Clinical Medicine, Linköping University, Linköping, Sweden; d Department of Oncology, Umeå University Hospital, Umeå, Sweden; e Department of Oncology and Pathology, Karolinska Institutet, Stockholm, Sweden; f Department of Oncology, Uppsala University Hospital, Uppsala, Sweden; g Department of Clinical Sciences Lund, Division of Oncology, Lund University, Lund, Sweden; h Department of Haematology, Oncology and Radiation Physics, Skåne University Hospital, Lund, Sweden; i Department of Clinical Sciences Lund, Division of Surgery, Lund University, Lund, Sweden; j Department of Biomedical and Clinical Medicine and Department of Oncology, Linköping University, Linköping, Sweden

## Abstract

**Background:**

Tamoxifen is an established treatment for breast cancer, but its long-term effects on survival and on secondary cancers are not fully evaluated.

**Material and methods:**

We studied 30 years outcome of 4124 postmenopausal patients who were randomized to receive (totally) two or five years of adjuvant tamoxifen.

**Results:**

After 5 years of follow-up, when tamoxifen treatment was finished in both groups, until 15 years of follow-up, overall mortality (HR 0.80, 95% CI 0.72–0.90, p < 0.001), breast cancer mortality for all patients (HR 0.80, 95% CI 0.68–0.94, p = 0.006) and breast cancer mortality for patients with estrogen receptor positive disease (HR 0.67, 95% CI 0.55–0.83, p < 0.001) were significantly reduced in the five-year group as compared to the two-year group. After 15 years, the difference remained but did not further increase.

In the five-year group, the incidence of contralateral breast cancer was gradually reduced during the entire period of observation. The incidence of lung cancer was also reduced in the five-year group. In contrast there was an increased endometrial cancer incidence in the five-year group and for those receiving 40 mg of tamoxifen this incidence was further increased.

**Conclusion:**

Three more years of tamoxifen therapy reduced the risk of breast cancer mortality. The difference was established during the first 15 years after randomization. Moreover, the incidence of contralateral breast cancer gradually decreased for 30 years. The incidence of lung cancer was reduced in the five-year group. In contrast the incidence of endometrial cancer was increased.

## Highlights

• After 30 years of follow-up, 5 years as compared to 2 years of tamoxifen.
• Had reduced breast cancer mortality.
• Had reduced all-cause mortality.
• Had reduced the incidence of contralateral disease.
• Had increased the incidence of endometrial cancer.

## 1 Introduction

Tamoxifen is an effective adjuvant treatment for early breast cancer reducing recurrence and mortality rates [1]. The Early Breast Cancer Trialists’ Collaboration Group (EBCTCG) overview, analyzing the benefit of 5 years of tamoxifen compared to no endocrine therapy, reported during the first 10 years after surgery, a significantly reduced recurrence rate for tamoxifen treated patients with estrogen receptor (ER+) positive tumors: year 0–4: Rate Ratio (RR) = 0.53; Standard Error (SE) 0.03; and year 5–9: RR = 0.68; SE 0.06, but suggested no further effect after year 10 (years 10–14: RR = 0.97; SE 0.10) [1]. The Swedish breast cancer, group trial comparing two and five years of adjuvant therapy recruited 4610 patients between 1983 and 1992. When data from the trial were first published in 1996 it was found that patients assigned to receive five years of tamoxifen, compared with two years of tamoxifen, experienced improvements in event free survival and overall survival [2]. These results changed clinical practice, and five years of adjuvant endocrine therapy became standard of care. The trial also contributed with data to several EBCTCG overviews [1,3]. These have confirmed an important carry over effect much beyond the duration of the tamoxifen medication. We here report 30 years follow-up of our trial.

As first demonstrated by Fornander and coworkers, tamoxifen increased the risk of endometrial cancer [4]. Moreover, it reduced the risk of contralateral breast cancer [5], and as we previously reported from the comparison of two and five years of tamoxifen therapy, it tended to reduce the incidence of lung cancer [6].

Estrogen receptor positive breast cancer is associated with late recurrences and prolonged follow-up from breast cancer trials is needed to fully evaluate benefits and side effects of new treatment regimes. Here we report results with 30 years follow-up on survival and incidence of contralateral breast cancer, endometrial cancer and lung cancer from the Swedish breast cancer group comparison of two years and five years adjuvant therapy.

### 1.1 Materials and methods

The trial was planned and organized by the Swedish Breast Cancer Group and involved five regional breast cancer centers and was approved by their Ethics Committees. Study characteristics by trial center are shown in Table 1.

**Table 1:** Study characteristics by trial center.

Trial center	Randomly assigned	Stage at diagnosis	Estrogen receptor status	Daily tamoxifen dose, mg
South Sweden	1320	II	Any	20
South-East Sweden, stage I	524	I	Positive	40
South-East Sweden, stage II-IIIA	1167	II-IIIA	Any	40
Stockholm	811	I-IIIA	Any	40
Uppsala-Örebro	583	II	Any	20
North Sweden	205	Node positive	Any	40

**Fig. 1:**
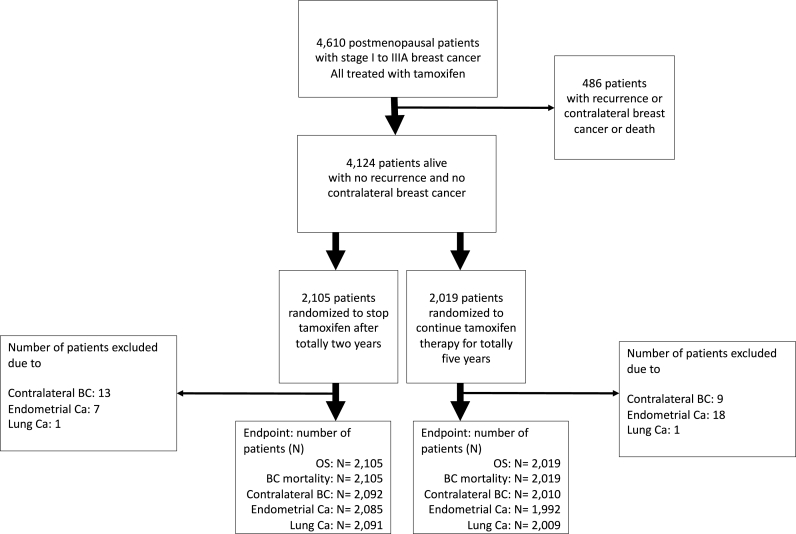
Consort diagram.

During the period 1983–1992 a total of 4610 postmenopausal women younger than 75 years with stage I to IIIA invasive breast cancer were entered into a randomized trial comparing five and two years of adjuvant tamoxifen. Mean age at treatment start was 62.7 years in the two-year group and 62.6 in the five-year group. Twenty milligrams daily doses were used at two centers, and 40 mg daily doses at the remaining three centers. The treatment was given regardless of ER status but most tumors had ER determined with isoelectric focusing or Abbot enzyme immunoassay as previously described [2]. Of these patients 4124 were alive, had no recurrence and no contralateral breast cancer two years after surgery, and contributed with information to comparisons between two-year (n = 2105) and five-year (n = 2019) of tamoxifen therapy. Of the 4124 patients 3102 were tested for ER-status and among those 2481 had ER positive disease. (see Fig. 1) Data on new cancers including contralateral breast cancer were obtained from the Swedish Cancer Register and data on survival from the Swedish Population Register [7].

### 1.2 Statistical analyses

Time for follow-up was defined as the time from randomization until the first event, death, or last observation (December 31, 2019). Median time for follow up was 30.2 years for living patients and 17.4 years for all patients. We examined crude cumulative incidence rates which is failure probabilities for a particular type of event, in the presence of other events, which may impede the event of interest to occur [8]. Breast cancer recurrence, contralateral breast cancer and death due to any cause were considered as competing events in the analyses of incidence of a new primary cancer. Data on contralateral breast cancer, cause of death and date of death, was available until year 2019 and breast cancer recurrence until 2003. The primary endpoint was time to death.

Subhazard ratios (SHR) and the 95% confidence intervals (CI) were estimated based on Fine and Gray's proportional subhazards model. For each analysis, cause-specific hazards (HR) and 95% CI based on Cox regression model were also calculated, censoring for cases representing the competing events above. The HRs and SHRs were similar, and both are reported in the tables. Analyses were done by the intention-to-treat, and the tests were two-sided.

A p value of 0.05 was considered to be statistically significant. The statistical analyses were performed using STATA/SE 13.1 [9].

## 2 Results

### 2.1 Mortality

**Fig. 2:**
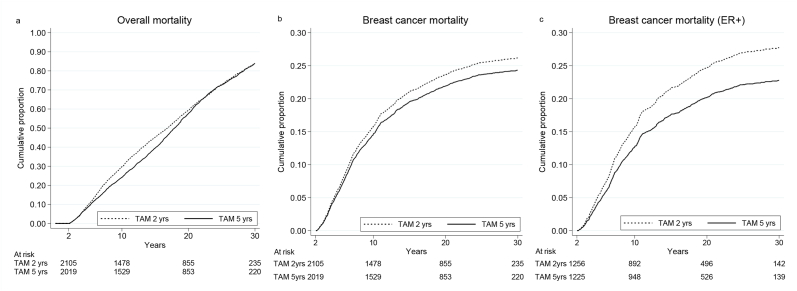
**a** left panel Overall mortality for patients randomized to two versus five years of tamoxifen. **b** middle panel Breast cancer mortality for all patients randomized. **c** right panel Breast cancer mortality for patients with estrogen receptor positive tumors.

Fig. 2 and Table 2, Table 3, Table 4 show mortality in the two- and five-year group respectively. After 5 years of follow-up, when all patients had finished tamoxifen treatment, until 15 years of follow-up, overall mortality (HR 0.80, 95% CI 0.72–0.90, p < 0.001) (Fig. 2a, Table 2), breast cancer mortality for all patients (HR 0.80, 95% CI 0.68–0.94, p = 0.006) (Fig. 2b, Table 3) as well as breast cancer mortality for patients with ER + tumors (HR 0.67, 95% CI 0.55–0.83, p < 0.001) (Fig. 2c, Table 4) were significantly decreased in the five-year group. After 15 years we did not observe any further difference in mortality between the treatment groups, not even for patients with tumors known to be ER+.

**Table 2:** Overall mortality. Events after 2 versus 5 years of tamoxifen treatment.

Years after surgery	TAM 2 yrs (n = 2105)	TAM 5 yrs (n = 2019)	HR 5 vs 2 yrs	95% CI
	Number of events			
≥2	1826	1744	0.94	0.88–1.01
2–5	211	180	0.88	0.72–1.07
5–15	741	617	0.80	0.72–0.90
15 –	874	947	1.08	0.98–1.18

CI = confidence interval; HR = hazard ratio; TAM = tamoxifen.

**Table 3:** Breast cancer mortality. Events after 2 versus 5 years of tamoxifen treatment.

Years after surgery	TAM 2 yrs (n = 2105)	TAM 5 yrs (n = 2019)	HR 5 vs 2 yrs	95% CI	SHR 5 vs 2 yrs	95% CI
	Number of events					
≥2	568	515	0.89	0.79–1.00	0.92	0.81–1.03
2–5	133	117	0.91	0.71–1.16	0.91	0.71–1.17
5–15	341	283	0.80	0.68–0.94	0.82	0.70–0.96
15 –	94	115	1.20	0.92–1.58	1.13	0.86–1 .49

CI = confidence interval; HR = hazard ratio; SHR = subhazard ratio; TAM = tamoxifen.

**Table 4:** Breast cancer mortality for patients with ER-positive tumors. Events after 2 versus 5 years of tamoxifen treatment.

Years after surgery	TAM 2 yrs (n = 1256)	TAM 5 yrs (n = 1225)	HR 5 vs 2 yrs	95% CI	SHR 5 vs 2 yrs	95% CI
	Number of events					
≥2	361	303	0.77	0.66–0.90	0.79	0.68–0.93
2–5	70	61	0.85	0.60–1.20	0.85	0.60–1.20
5–15	222	164	0.67	0.55–0.83	0.68	0.56–0.84
15 –	69	78	1.01	0.73–1.39	0.99	0.71–1.37

CI = confidence interval; HR = hazard ratio; SHR = subhazard ratio; TAM = tamoxifen.

### 2.2 Contralateral breast cancer

**Fig. 3:**
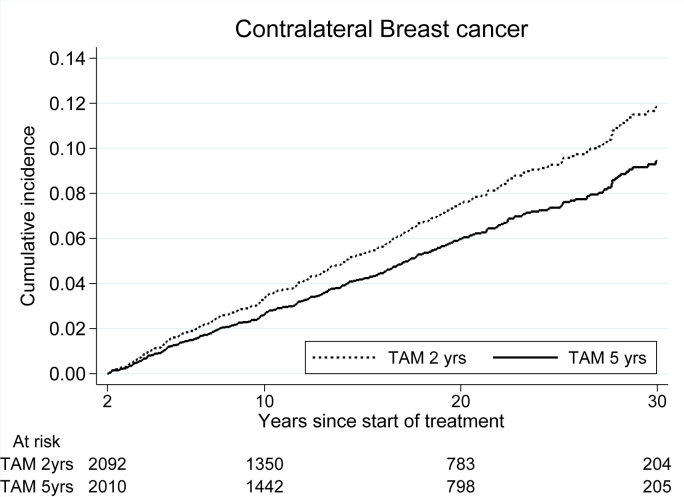
Incidence of contralateral breast cancer for patients randomized to two versus five years of tamoxifen.

The incidence of contralateral breast cancer was reduced in the five-year group, HR 0.75 (95% CI 0.59–0.95, p = 0.029) as illustrated in Table 5 and Fig. 3. Comparing patients receiving 20 mg or 40 mg of tamoxifen daily, we observed no difference in the incidence of contralateral breast cancer, HR 1.02 (95% CI 0.80–1.30, p = 0.87).

**Table 5:** Incidence of contralateral breast cancer. Events after 2 versus 5 years of tamoxifen treatment.^a^

Years after surgery	TAM 2 yrs (n = 2092)	TAM 5yrs (n = 2010)	HR 5 vs 2 yrs	95% CI	SHR 5 vs 2 yrs	95% CI
	Number of events					
≥2	158	123	0.75	0.59–0.95	0.79	0.62–1.00
2–5	29	21	0.74	0.42–1.30	0.74	0.42–1.31
5–15	72	51	0.67	0.47–0.96	0.69	0.48–0.99
15 –	57	51	0.86	0.59–1.26	0.86	0.59–1.26

CI = confidence interval; HR = hazard ratio; SHR = subhazard ratio; TAM = tamoxifen.

a 13 patients in the treatment group of 2 years TAM and 9 patients in the treatment group of 5 years TAM were diagnosed with contralateral breast cancer before the randomization. These patients were excluded in this analysis.

### 2.3 Endometrial cancer

**Fig. 4:**
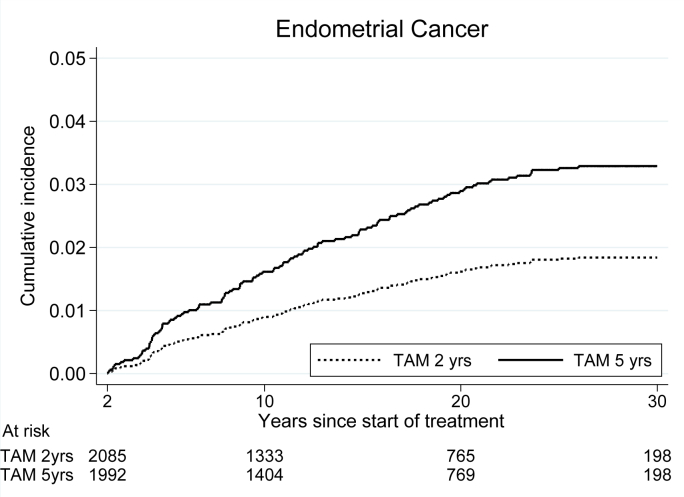
Incidence of endometrial cancer for patients randomized to two versus five years of tamoxifen.

In the five-year group, the incidence of endometrial cancer was increased compared with the two-year group, HR 1.65 (95% CI 1.12–2.42, p = 0.010), Table 6 and Fig. 4. During the three extra years of tamoxifen therapy the incidence was markedly increased, HR 3.49 (CI 1.40–8.71, p = 0.007). Seventy endometrial cancers were diagnosed in the five-year group as compared with 42 in the two-year group. Beyond 15 years there were 18 endometrial cancers both in the five-year and the two-year group.

**Table 6:** Incidence of endometrial cancer. Events after 2 versus 5 years of tamoxifen treatment.^a^

Years after surgery	TAM 2 yrs (n = 2085)	TAM 5 yrs (n = 1992)	HR 5 vs 2 yrs	95% CI	SHR 5 vs 2 yrs	95% CI
	Number of events					
≥2	42	70	1.65	1.12–2.42	1.81	1.23–2.66
2–5	6	20	3.49	1.40–8.71	3.54	1.42–8.82
5–15	18	32	1.69	0.95–3.01	1.93	1.07–3.47
15 –	18	18	1.00	0.52–1.93	0.96	0.49–1.85

CI = confidence interval; HR = hazard ratio; SHR = subhazard ratio; TAM = tamoxifen.

a 22 patients were diagnosed with contralateral breast cancer before the randomization. Furthermore, 7 patients in the treatment group of 2 years TAM and 18 patients in that of 5 years TAM were diagnosed with endometrial cancer before (the) randomization. These patients were excluded in this analysis.

The risk to develop endometrial cancer tended to be higher among patients receiving 40 mg tamoxifen compared to those receiving 20 mg, HR 1.44 (95% CI 0.96–2.61, p = 0.079).

### 2.4 Lung cancer

**Fig. 5:**
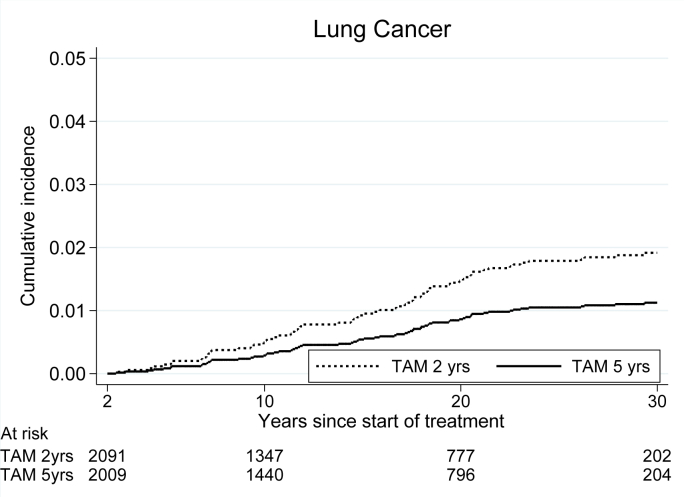
Incidence of primary lung cancer for patients randomized to two versus five years of tamoxifen.

In agreement with our previous report from this trial^6^, we found a lower incidence of lung cancer in the five-year group, HR 0.51 (95% CI 0.32–0.84, p = 0.008). The present data with prolonged follow-up showed that the decreased incidence is seen during the first fifteen years of follow-up (Fig. 5 and Table 7). The effect on lung cancer incidence was similar for the groups receiving 20 mg respectively 40 mg of tamoxifen, HR = 0.85 (95% CI 0.53–1.37, p = 0.51). With the analysis confined to small cell lung cancer and squamous cell lung cancer (25 patients for the whole period), the lung cancer incidence was markedly decreased in the five-year group, HR 0.22 (95% CI 0.08–0.59, p = 0.003).

**Table 7:** Incidence of lung cancer. Events after 2 versus 5 years of tamoxifen treatment. a

Years after surgery	TAM 2 yrs (n = 2091)	TAM 5yrs (n = 2009)	HR 5 vs 2 yrs	95% CI	SHR 5 vs 2 yrs	95% CI
	Number of events					
≥2	46	25	0.51	0.32–0.84	0.58	0.35–0.97
2–5	6	2	0.34	0.07–1.69	0.25	–
5–15	23	6	0.24	0.10–0.59	0.28	0.11–0.70
15 –	17	17	0.95	0.49–1.87	0.91	0.46–1.80

CI = confidence interval; HR = hazard ratio; SHR = subhazard ratio; TAM = tamoxifen.

a Before the randomization, 22 patients were diagnosed with contralateral breast cancer and 1 patient in each treatment group were diagnosed with lung cancer. These patients were excluded in this analysis.

## 3 Discussion

In the present study with extended follow-up data, overall mortality, cumulative breast cancer mortality, incidence of contralateral breast cancer and incidence of lung cancer were delayed in the five year-group as compared to the two year-group. It is well known that the effect of tamoxifen remains beyond the duration of therapy. This continued reduction of breast cancer events after discontinuation of treatment is referred to as the tamoxifen carry over effect. In the ATLAS trial, comparing 5 vs. 10 years of tamoxifen, the benefit of extended therapy was not evident until after year 10; 5–9 years: RR = 0.90 (95% CI 0.79–1.02) and ≥10 years: RR = 0.75 (0.62–0.90) and the aTTom trial provided similar results [[10], [11], [12]]. The study of Ekholm [13] and coworkers comparing 2 years of tamoxifen therapy of premenopausal patients with control reported three decades of follow-up. The carry over effect tended to increase with time. The mortality hazard ratios were 0.67 the first five years, 0.60 during 5–15 years and 0.53 for 15–30 years of follow-up.

In this study, the difference in breast cancer mortality rates between the five year and two-year groups of patients with ER + disease increased until 15 years after surgery but not beyond 15 years. It is possible that for endocrine sensitive tumors inducing late recurrence, most of the tamoxifen effect was achieved after only two years. Furthermore, late recurring tumors may have been effectively controlled by aromatase inhibitors, reintroduction of tamoxifen or other types of systemic treatment controlling metastases and decreasing the risk of breast cancer death. Moreover, there are patients identified with molecular tools that have excellent long-term survival without endocrine treatment [14,15].

With contralateral breast cancer as endpoint, our data do support a prolonged carry over effect. For endometrial cancers beyond 15 years there were 18 cases both in the five-year and the two-year group. Thus, there was no clear evidence for a prolonged carry over effect for the induction of endometrial cancer, but postmenopausal patients should be informed about the increased risk during and shortly after tamoxifen medication.

We used two different non-randomized tamoxifen doses 20 or 40 mg daily and found no difference in incidence of contralateral breast cancers between the patients receiving 20 or 40 mg. However, the incidence of endometrial cancer tended to be increased among patients receiving 40 mg daily. The optimal dose for tamoxifen as adjuvant therapy has not been established but our data do not support an increase to 40 mg daily for postmenopausal patients.

The results in this report could be compared to those comparing two years of tamoxifen with a control group [4,16]. The most benefit from adjuvant tamoxifen is achieved during the initial two years of therapy. Patients with low risk of recurrence, for whom the standard duration of five years endocrine therapy would be overtreatment, could be identified with molecular markers [17,18]. Patients need careful advice and follow-up to be able to balance benefit from continued endocrine therapy with side effects.

In accordance with the ATAC trial where 51 lung cancers were reported in the anastrozole group as compared with 34 in the tamoxifen group, we found fewer lung cancers in the five-year group [19]. Also, outside the framework of clinical trials there are reports of reduced numbers of lung cancers among tamoxifen treated breast cancer patients [20,21]. Moreover, other reports describe an increased number of lung cancers among estrogen replacement treated women [22] and experimental evidence demonstrating antiestrogen inhibition of lung cancer cell growth [23,24]. This motivates further study of lung cancers in trials performed with tamoxifen, raloxifene and aromatase inhibitors. It is important to consider the possibility to misclassify a lung metastasis from breast cancer as primary lung cancer and that the decreased number of lung cancers in the five-year group could be explained by improved control of metastatic disease. Importantly, the number of squamous cell and small cell lung cancers which are histologically clearly distinguished from breast cancer were reduced in the five-year group in the present study. This supports a real effect rather than misclassification. Antiestrogens may possibly have a future role in the prevention of lung cancer.

The trial described above contributed to the change of clinical practice to five rather than two years of adjuvant endocrine therapy. Three extra years of tamoxifen therapy gradually reduced the risk of breast cancer mortality. In our current studies of the trial we aim to identify the subgroups of patients that do need five years of therapy as well as those for which a reduction to two years is enough to control the disease and reduce side effects.

## Funding

The study was supported by grants without numbers from the King Gustaf the Vth Jubilee Clinic Cancer Foundations in Gothenburg and from the Swedish governmental grants to scientists working in health care (ALF), the 10.13039/501100002794Swedish Cancer Society, the Swedish Breast Cancer Association, Alice Swenzon Foundation, Walter Anderssons Foundation, Onkologiska Klinikerna i Linköping Forskningsfond, Ulla and Tibor Veres award. The funders have no role in the study design, data collection, analysis or interpretation of the data or writing of the report.

## Declaration of interest statement

The authors have declared no conflicts of interest.

## Ethical approval

The present study of long-term effects of tamoxifen treatment has been carried out with the approval of the Regional Ethical Review Board in Linköping Sweden (Dnr 2011/317-31).

## Authors’ contributions

A.N. wrote most of the manuscript, H.F. performed statistical analyses and prepared figures and tables in close collaboration with O.S., B.N. and A.W. planned the clinical trial with B.N. serving as PI. J.R. kept the database, T.F., T.H., H.L., NO.B. and P.M. were responsible for patient recruitment at their trial centers and L.R. coordinated discussions regarding data management and endpoints. All authors reviewed the manuscript.

## Abbreviations

CI: confidence interval
ER: estrogen receptor
ER+: estrogen receptor positive
HR: hazard ratio
SHR: subhazard ratio
TAM: tamoxifen

## References

[1] Early Breast Cancer Trialists' Collaborative G., Davies C., Godwin J., Gray R., Clarke M., Cutter D. (2011). Relevance of breast cancer hormone receptors and other factors to the efficacy of adjuvant tamoxifen: patient-level meta-analysis of randomised trials. Lancet.

[2] (1996). Randomized trial of two versus five years of adjuvant tamoxifen for postmenopausal early stage breast cancer. Swedish Breast Cancer Cooperative Group. J Natl Cancer Inst.

[3] Early Breast Cancer Trialists' Collaborative G. (2005). Effects of chemotherapy and hormonal therapy for early breast cancer on recurrence and 15-year survival: an overview of the randomised trials. Lancet.

[4] Fornander T., Rutqvist L.E., Cedermark B., Glas U., Mattsson A., Silfversward C. (1989). Adjuvant tamoxifen in early breast cancer: occurrence of new primary cancers. Lancet.

[5] Ryden S., Ferno M., Moller T., Aspegren K., Bergljung L., Killander D. (1992). Long-term effects of adjuvant tamoxifen and/or radiotherapy. The south Sweden breast cancer trial. Acta Oncol.

[6] Rosell J., Nordenskjold B., Bengtsson N.O., Fornander T., Hatschek T., Lindman H. (2017). Long-term effects on the incidence of second primary cancers in a randomized trial of two and five years of adjuvant tamoxifen. Acta Oncol.

[7] https://www.socialstyrelsen.se/statistik-och-data/register/dodsorsaksregistret/.

[8] Marubini E., Valsecchi M., V B. (1995). Statistics in practice: analalysing survival data from clinical trials and obserational studies.

[9] (2013). StataCorp. Stata statistical software: release 13 collage.

[10] Davies C., Pan H., Godwin J., Gray R., Arriagada R., Raina V. (2013). Long-term effects of continuing adjuvant tamoxifen to 10 years versus stopping at 5 years after diagnosis of oestrogen receptor-positive breast cancer: ATLAS, a randomised trial. Lancet.

[11] Gray R.G.R.D., Handley K. (2013). aTTom: long-term effects of continuing adjuvant tamoxifen to 10 years versus stopping at 5 years in 6953 women with early breast cancer. J Clin Oncol.

[12] Bartlett J.M.S., Sgroi D.C., Treuner K., Zhang Y., Ahmed I., Piper T. (2019). Breast Cancer Index and prediction of benefit from extended endocrine therapy in breast cancer patients treated in the Adjuvant Tamoxifen-To Offer More? (aTTom) trial. Ann Oncol : Off J European Soc Med Oncol / ESMO..

[13] Ekholm M., Bendahl P.O., Ferno M., Nordenskjold B., Stal O., Ryden L. (2016). Two years of adjuvant tamoxifen provides a survival benefit compared with No systemic treatment in premenopausal patients with primary breast cancer: long-term follow-up (> 25 years) of the phase III SBII:2pre trial. J Clin Oncol.

[14] Sjostrom M., Chang S.L., Fishbane N., Davicioni E., Hartman L., Holmberg E. (2020). Comprehensive transcriptomic profiling identifies breast cancer patients who may Be spared adjuvant systemic therapy. Clin Cancer Res : an Off J Am Assoc Cancer Res.

[15] Esserman L.J., Yau C., Thompson C.K., van 't Veer L.J., Borowsky A.D., Hoadley K.A. (2017). Use of molecular tools to identify patients with indolent breast cancers with ultralow risk over 2 decades. JAMA Oncol.

[16] Rutqvist L.E., Johansson H., Stockholm Breast Cancer Study G. (2007). Long-term follow-up of the randomized Stockholm trial on adjuvant tamoxifen among postmenopausal patients with early stage breast cancer. Acta Oncol.

[17] Jerevall P.L., Ma X.J., Li H., Salunga R., Kesty N.C., Erlander M.G. (2011). Prognostic utility of HOXB13:IL17BR and molecular grade index in early-stage breast cancer patients from the Stockholm trial. Br J Cancer.

[18] Parker J.S., Mullins M., Cheang M.C., Leung S., Voduc D., Vickery T. (2009). Supervised risk predictor of breast cancer based on intrinsic subtypes. J Clin Oncol.

[19] Cuzick J., Sestak I., Baum M., Buzdar A., Howell A., Dowsett M. (2010). Effect of anastrozole and tamoxifen as adjuvant treatment for early-stage breast cancer: 10-year analysis of the ATAC trial. Lancet Oncol.

[20] Bouchardy C., Benhamou S., Schaffar R., Verkooijen H.M., Fioretta G., Schubert H. (2011). Lung cancer mortality risk among breast cancer patients treated with anti-estrogens. Cancer.

[21] Chu S.C., Hsieh C.J., Wang T.F., Hong M.K., Chu T.Y. (2017). Antiestrogen use in breast cancer patients reduces the risk of subsequent lung cancer: a population-based study. Cancer Epidemiol.

[22] Greiser C.M., Greiser E.M., Doren M. (2010). Menopausal hormone therapy and risk of lung cancer-Systematic review and meta-analysis. Maturitas.

[23] Garon E.B., Pietras R.J., Finn R.S., Kamranpour N., Pitts S., Marquez-Garban D.C. (2013). Antiestrogen fulvestrant enhances the antiproliferative effects of epidermal growth factor receptor inhibitors in human non-small-cell lung cancer. J Thorac Oncol.

[24] Dubois C., Rocks N., Blacher S., Primac I., Gallez A., Garcia-Caballero M. (2019). Lymph/angiogenesis contributes to sex differences in lung cancer through oestrogen receptor alpha signalling. Endocr Relat Cancer.

